# Surgical Outcomes of Gonioscopy-Assisted Transluminal Trabeculotomy (GATT) in Primary and Secondary Open- and Closed-Angle Glaucoma

**DOI:** 10.3390/diagnostics15101226

**Published:** 2025-05-13

**Authors:** Liron Naftali Ben Haim, Veronika Yehezkeli, Assaf Kratz, Nimrod Dar, Tal Sharon, Gal Harel, Zvia Burganski-Eliash, Avner Belkin

**Affiliations:** 1 Meir Medical Center, Kfar Saba 4428164, Israel; lironbh4690@gmail.com (L.N.B.H.);; 2 The Sackler Faculty of Medicine, Tel Aviv University, Tel Aviv 39040, Israel; 3 Soroka Medical Center, Beer Sheva 84101, Israel; 4 Faculty of Health Sciences, Ben Gurion University of the Negev, Beer Sheva 84101, Israel; 5 Goldschleger Eye Institute, Sheba Medical Center, Ramat Gan 52620, Israel

**Keywords:** trabeculotomy, glaucoma, goniotomy, combined surgery, minimally invasive glaucoma surgery

## Abstract

**Background:** Gonioscopy-assisted transluminal trabeculotomy (GATT) is a minimally invasive, ab interno conjunctival-sparing glaucoma surgery aimed at the trabecular meshwork and the inner wall of Schlemm’s canal. The goal of this study is to report on the success of GATT in a large group of patients with a wide variety of open- and closed-angle glaucomas with or without cataract extraction and to report on risk factors for failure. **Methods:** A retrospective chart review of consecutive patients with primary or secondary open- or closed-angle glaucoma who underwent GATT, with or without concomitant phacoemulsification. Demographics, baseline clinical characteristics, and postoperative outcomes were collected from patients’ medical records. Primary outcomes were success rates (IOP of 18 mmHg or lower and one of the following: IOP reduction > 30% from baseline on the same or fewer medications or an IOP ≤ baseline with fewer medications as compared to baseline) and complication rates. Intraocular pressure (IOP) and the number of glaucoma medications were secondary outcome measures. **Results:** GATT was performed on 126 eyes of 121 patients. Mean follow-up was 583 ± 266 days. Cumulative success at 1Y was 0.88 for GATT combined with cataract extraction, 0.96 for GATT alone, 0.88 for primary open-angle glaucoma (POAG), 0.89 for secondary open-angle glaucoma (SOAG), and 0.76 for primary angle-closure glaucoma (PACG). IOP decreased from a mean of 20.65 mmHg to 14.1 mmHg, and medication decreased from a mean of 3.47 to 1.4 at the last follow-up. Forty-four eyes (34%) were classified as failures. Factors associated with an increased risk of failure were worse preoperative corrected visual acuity (OR = 2.46, *p* = 0.024) and a postoperative IOP spike (OR = 2.62, *p* = 0.028). Twelve eyes (9.5%) required further surgery for IOP control. Risk factors for requiring further surgery for IOP control were preoperative maximal IOP (OR = 1.066, *p* = 0.047) and a postoperative IOP spike (OR = 4.531, *p* = 0.036). **Conclusions:** GATT achieved good surgical success with good IOP and medication reduction across a wide range of glaucomas, in combination with lens extraction or as a standalone procedure. GATT should be considered early in the treatment paradigm of medically uncontrolled glaucoma.

## 1. Background

Gonioscopy-assisted transluminal trabeculotomy (GATT), originally described by Grover et al. in 2014 [1] is a minimally invasive, non-bleb-forming ab-interno procedure in which a trabeculotomy is performed using a prolene suture. In the years since its introduction, GATT has been shown to be effective in intraocular pressure (IOP) and medication reduction in a wide variety of primary and secondary open-angle glaucomas [2,3,4,5,6,7]. Of note is its efficacy in the following aggressive glaucomas: pseudoexfoliative, juvenile, uveitic, and in patients with a history of prior incisional surgery [8,9,10,11,12,13,14].

Mean IOPs at the end of follow-up in several large retrospective and prospective studies examining GATT have been shown to be in the low teens [7,13,15,16,17,18,19] which is a target pressure suitable for many (though not all) glaucoma patients, even in those with moderate or advanced disease.

An important advantage of GATT is that it spares the conjunctiva, thus leaving room for subconjunctival filtering procedures in the future. This is an important feature in the treatment of a life-long chronic disease, especially considering that subconjunctival filtering procedures have significant failure rates after a few years: the authors of the Tube versus Trabeculectomy study reported a cumulative failure rate for trabeculectomy of 46.9% at 5 years, and a recent randomized controlled study comparing trabeculectomy with an ab externo MicroShunt found 29.9% failure of trabeculectomy at 1 year [20,21]. It is this fact, along with a favorable efficacy and safety profile, that suggests a place for GATT early in the surgical treatment paradigm of glaucoma, prior to engaging the subconjunctival space. However, GATT is not suitable for all patients and has a unique adverse events profile. Finding the appropriate place for GATT in the treatment paradigm for glaucoma is an ongoing challenge, as is the understanding of which patients stand to benefit most from it.

The goal of this study is to report on the outcomes of GATT in a relatively large group with a wide variety of open- and closed-angle glaucomas with or without cataract extraction and to search for risk factors for failure and need for reoperation.

## 2. Methods

A retrospective chart review was conducted of consecutive patients with primary or secondary open- or closed-angle glaucoma who underwent GATT with or without concomitant phacoemulsification between October 2019 and October 2021. Two glaucoma specialists and 2 glaucoma fellows performed all surgeries at one site. All surgeries were performed from a temporal approach. Patients with ocular hypertension (OHTN) primary angle closure glaucoma (PACG) and primary or secondary open-angle glaucoma (POAG and SOAG) underwent a standalone GATT (solo-GATT), GATT combined with phacoemulsification cataract surgery (phaco-GATT), GATT combined with phacoemulsification cataract surgery and goniosynechialysis (phaco-GSL-GATT) or GATT with intra-ocular lens (IOL) fixation following IOL subluxation.

The study was approved by the human research committee in accordance with the Declaration of Helsinki. An institutional review board (IRB)/ethics committee approval has been obtained. We included patients with a diagnosis of glaucoma or ocular hypertension (OHT). The diagnosis of glaucoma was based on the presence of ophthalmoscopically detectable glaucomatous optic neuropathy with correlating visual field defects. Ocular hypertension was defined as IOP ≥ 22 mmHg without glaucomatous optic neuropathy. The indication for surgery in ocular hypertensive (OHT) patients was an IOP above target on maximally tolerated medication.

Patients with a follow-up of less than three months (unless they underwent reoperation) were excluded. A comprehensive ocular examination, including gonioscopy and dilated fundus examination, was performed for all patients before surgery.

### 2.1. Surgical Technique

As described previously, Ref. [1] the procedure began with the administration of topical anesthesia and 5% povidone–iodine drops, followed by the creation of one or two clear corneal paracenteses. Lidocaine 1% was instilled into the anterior chamber followed by a cohesive viscoelastic. A thermally blunted 5-0 Prolene suture was introduced into the anterior chamber. The patient’s head was tilted away from the surgeon while the microscope was tilted as well to optimize visualization. A small nasal goniotomy was created using an MVR blade through a 2.2 mm temporal corneal incision. The Prolene suture was inserted into Schlemm’s canal and advanced circumferentially according to the desired extent of the trabeculotomy. The suture was then internalized through the inner wall of Schlemm’s canal and the trabecular meshwork into the anterior chamber. The resulting hyphema and viscoelastic material were thoroughly irrigated using an anterior chamber maintainer. At the end of surgery, topical antibiotics and steroids were administered. In cases of angle closure, the anterior chamber was first deepened using an ophthalmic viscoelastic device (OVD). Goniosynechiolysis was performed when necessary to release peripheral anterior synechiae (PAS) and to expose the trabecular meshwork. In some cases, the crystalline lens was removed prior to GATT to gain easier access to the anterior chamber angle. For eyes with malignant glaucoma iridectomy, zonulohyaloidectomy, and vitrectomy (IZHV) was performed prior to GATT.

The extent of trabeculotomy was at the discretion of the surgeon and depended on the specific features of the patient’s disease, the indication for surgery, and intra-operative considerations.

### 2.2. Postoperative Protocol

Following surgery, all patients were instructed to use a combination of topical dexamethasone, neomycin, and polymyxin B (Maxitrol (dexamethasone, neomycin, and polymyxin B; Novartis Pharmaceuticals UK Ltd., Frimley, Camberley, UK)) administered six times daily for three days, three times daily for another three days, and then tapered off over a period of two to three weeks. In addition, topical nonsteroidal anti-inflammatory drug drops (Nevanac, 0.1% ophthalmic suspension, Alcon, Couvreur N.V., Puurs-Sint-Amands, Belgium) were applied three times each day for one month. In most cases the patient was advised to discontinue any pre-existing glaucoma medications. The decision whether and when to start IOP-lowering medications was at the surgeon’s discretion.

Follow-up was scheduled at postoperative day 1, day 3–4, week 1, week 3, week 6, month 3, and then every six months or sooner if necessary.

### 2.3. Outcome Measures

Demographics, baseline clinical characteristics, postoperative outcomes at each follow-up visit, and relevant surgical information (degrees and location of trabeculotomy and intraoperative complications) were collected from the patients’ medical records. Baseline IOPs and medication were recorded at the visit at which surgery was decided upon. The severity of glaucoma was defined according to mean deviation (MD) on Humphrey visual field 24-2 SITA-standard tests (mild: 0 to −5.99, moderate: −6 to −11.99, and severe: −12 or lower) [22]. The analysis of glaucoma type-OAG included POAG), juvenile open-angle glaucoma (JOAG), normal-tension glaucoma (NTG), and OHTN. Secondary open-angle glaucoma (SOAG) included pseudoexfoliative glaucoma (PXG), pigment dispersion glaucoma (PDG), and uveitic glaucoma. Primary angle closure glaucoma (PACG) included patients with PACG and with combined mechanism glaucoma (CMG) that included angle closure as part of the mechanism.

Primary outcomes were cumulative success and complication rates. IOP and the number of glaucoma medications were secondary outcome measures. Success criterion A was defined as an IOP of 18 mmHg or lower and one of the following: IOP reduction >30% from baseline on the same or fewer medications or an IOP ≤ baseline with fewer medications as compared to baseline. Failure at last follow up (FU) visit was defined as failing to meet these criteria, needing further IOP-lowering surgery, or loss of light perception. Success criterion B was defined according to the Primary TVT study as IOP greater than 21 mmHg or reduced by less than 20% from baseline, IOP of 5 mmHg or less, reoperation for glaucoma, loss of light perception vision [20].

An IOP spike was defined as an IOP > 30 mmHg or >10 mmHg above baseline IOP within 3 months of surgery. Hypotony was defined as IOP lower than 6 mmHg at any time, with or without signs of clinical hypotony. A hyphema was classified either as a macroscopic (layered) hyphema or a microscopic hyphema (more than +2). Finally, all intraoperative complications were documented.

### 2.4. Statistical Methods

The analysis was performed with SPSS-25 software (IBM, Armonk, NY, USA). Odds ratio analyses were performed using a logistic regression. Correlation analyses were performed using Pearson correlation. Data were expressed as numbers and percentage for qualitative variables and as mean and standard deviation for continuous parameters. Normality distribution was checked for continuous data (Shapiro–Wilk test), and tests were performed in accordance: T-test or Mann–Whitney non-parametric test. For nominal variables, the Chi-Square test was performed. Survival curves were plotted by the Kaplan–Meier method and compared using the log-rank test. A backwards stepwise multivariate analysis was performed, including all variables that were *p* < 0.05 in univariate analysis. In all analyses, a two-sided *p* < 0.05 was considered statistically significant.

## 3. Results

### 3.1. Patient Demographics

GATT was performed on 126 eyes of 121patients, of whom 58% were males. Mean age at the time of surgery was 68.9 ± 14.4. A 180-degree GATT (hemi-GATT) was performed in 39.7% of eyes; 65% of these were performed inferiorly. Eighty-seven (69.1%) eyes underwent combined surgery with phacoemulsification (phaco-GATT). The three most common types of glaucoma were PXG) (26%), POAG (25.2%), PACG (14.2%). Mean preoperative MD was −9.172 ± 6.68 (range (−)1 to (−)20), and forty-nine eyes (38.9%) had advanced glaucoma. Mean preoperative IOP was 20.65 ± 7.28 mmHg on a mean of 3.47 ± 1.54 medications. Mean follow-up was 583 ± 266 days. Baseline demographic characteristics are presented in Table 1.

### 3.2. Surgical Success-Criterion A

The Kaplan–Meier survival analysis was used to compare surgical success rates among patients with different glaucomas (POAG, SOAG, PACG) and among patients that underwent different procedures (phaco GATT vs. solo GATT). The 1-year success rates were 87.9% for POAG, 89.4% for SOAG, and 76.7% for PACG. At 2 years, the cumulative success rates were 67.8% for POAG, 66.0% for SOAG, and 47.8% for PACG.

At 1 year, the success rate for the phaco-GATT group was 88.9%, while the solo GATT group had a success rate of 96.4%. By 2 years, the cumulative success rates declined to 65.0% for phaco-GATT and 55.5% for solo GATT.

**Figure 1:**
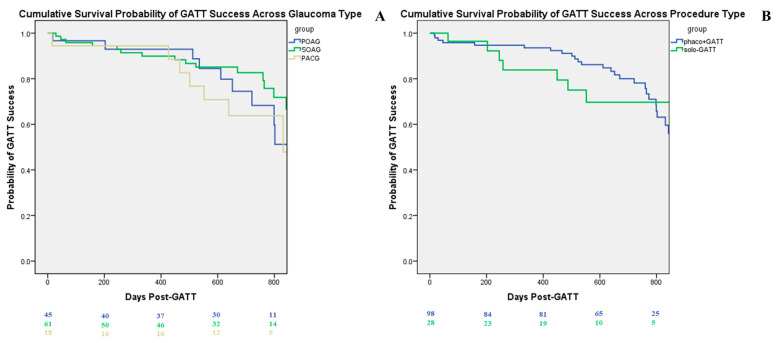
Survival curves criterion A. (**A**) Survival curve by glaucoma type, showing cumulative success at 800 days for POAG, SOAG, and PACG. (**B**) Survival curve by procedure type, showing cumulative success at 800 days for phaco-GATT and for solo GATT.

The log-rank test for both analyses demonstrated no statistically significant difference in survival distributions among the groups (Figure 1A,B).

### 3.3. Failure Criterion A

Forty-four eyes (34%) were classified as failures. Of these, 12 eyes required additional surgery (four trabeculectomies and eight Ahmed glaucoma valve implantations (AGVs)). Twenty-two eyes (17.4%) experienced IOP levels exceeding 18 mmHg, 26 eyes (20.6%) had IOP values higher than their baseline, and 6 eyes (4.7%) required additional medications compared to their baseline. Fourteen eyes had both IOP levels above 18 and IOP values higher than their baseline, thus meeting two failure criteria (Table 2).

### 3.4. Failure Criterion B

Fifty-five eyes (43%) were classified as failures at last FU. Failure was due to IOP > 21 in 10% of patients (*n* = 13) and due to less than 20% reduction from baseline IOP in 42% (53 patients).

### 3.5. Glaucoma Type

Table 3 summarizes the surgical outcomes of GATT across different glaucoma subtypes, highlighting success and failure rates (criterion A), IOP reduction, and changes in glaucoma medication use at last FU. The highest success rate was observed in OHTN (75%), followed by CMG (73.3%) and PXG(72.2%). PACG and POAG showed relatively lower success rates, at 55.5% and 58%, respectively. The IOP reduction percentage varied across subtypes, with OHTN showing the greatest reduction (44.6%), while PACG had the smallest reduction (5.5%). Changes in glaucoma medication use also differed, with OHTN patients experiencing the greatest reduction (100%).

### 3.6. Solo GATT

In our cohort, solo GATT was performed in 29 patients, with the largest subgroups being POAG (*n* = 7) and PXG (*n* = 9). The success rates were comparable between these two groups, with 57% (4/7) in POAG and 66% (6/9) in PXG at last FU.

### 3.7. Risk Factors for Failure

Univariate analysis revealed that pre-op logMAR VA and postoperative IOP spikes were associated with failure to achieve criterion A. In a multivariate logistic regression model, both factors were found to be significant predictors. Better preoperative visual acuity was associated with a higher likelihood of success (OR = 0.406, 95% CI: 0.185–0.889, *p* = 0.024), indicating that patients with better baseline vision were more likely to achieve favorable surgical outcomes. Conversely, the occurrence of a postoperative IOP spike significantly reduced the odds of surgical success (OR = 0.381, 95% CI: 0.162–0.900, *p* = 0.028). The univariate analysis is shown on Table S1.

### 3.8. Risk Factors for Re-Operation

Four factors were associated with an increased risk of failure requiring reoperation for IOP reduction: preoperative maximal IOP (*p* = 0.003), preoperative total number of medications (*p* = 0.01), preoperative oral CAI usage (*p* = 0.04), and the occurrence of a postoperative IOP spike (*p* = 0.008).

In a multivariate logistic regression model, higher postoperative peak IOP (TMAX) and IOP spikes were both independently associated with the need for additional glaucoma surgery after GATT. Each 1 mmHg increase in TMAX increased the odds of requiring another procedure by 6.6% (OR = 1.066, *p* = 0.047). Additionally, patients who experienced an IOP spike were 4.5 times more likely to need another surgery (OR = 4.531, *p* = 0.036). Age at surgery, gender, and postoperative macro-hyphema were not found to be associated with failure requiring reoperation. The type of glaucoma was not correlated to the failure rate (Table S2).

### 3.9. IOP and Medications

IOP decreased from a preoperative mean of 20.65 ± 7.28 mmHg (range 10–48 mmHg) to 14.1 ±6.4 mmHg (range 6–44 mmHg) atfinal FU.

**Figure 2:**
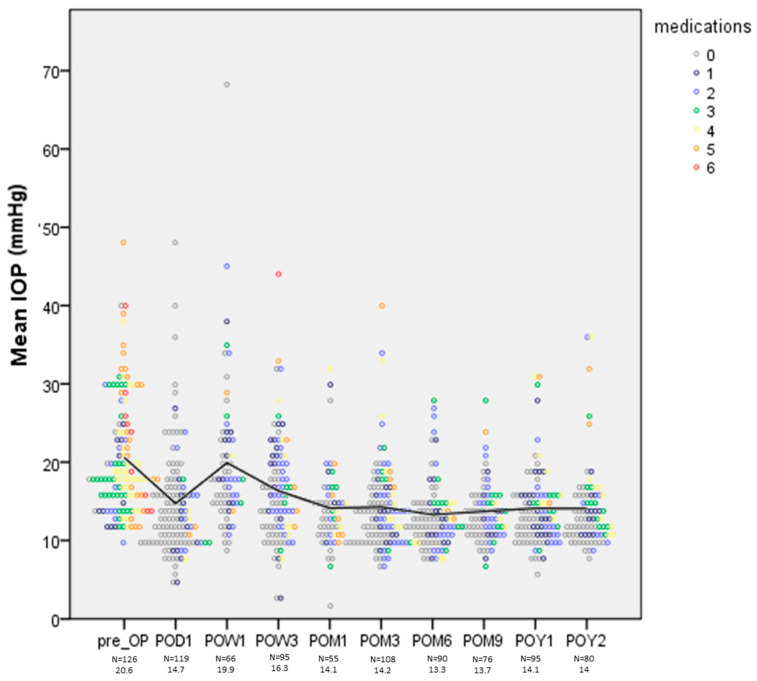
IOP medication for all patients during the study period. The color-coded dots represent the number of medications for individual patients.

**Figure 3:**
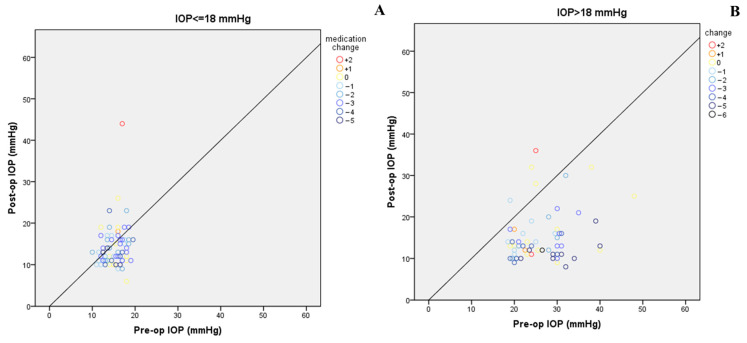
Scatter plots of pre- and postoperative IOPs and medication usage in patients with a baseline IOP of under 18 mmHg (**A**) and 18 mmHg or above (**B**).

Medication decreased from a mean of 3.47 ± 1.54 to 1.4 ± 1.5 at last FU. IOP decreased by 27% and 35.4% in POAG and SOAG, respectively. The number of medications decreased from an average of 3.3 ± 1.6 to 1.1 ± 1.6 in POAG, and from an average of 3.7 ± 1.5 to 1.5 ± 1.4 in SOAG. Figure 2 shows mean IOPs during follow-up, together with individual pre- and postoperative IOPs and medications. Scatter plots of pre- and postoperative IOPs and medications for patients with a baseline IOP of over or under 18 mmHg are shown in Figure 3A,B. In patients in the latter group, IOP was reduced from a mean of 15.3 mmHg to 14.3 mmHg (*p* = 0.2) and medications were reduced from 3.3 ± 1.3 to 1.3 ± 1.5 (*p* = 0.001).

Table 4 shows pre- and postoperative IOP and medications for all follow-up visits for three groups—phaco-GATT for OAG, solo GATT for OAG, and GATT combined with cataract extraction for PACG (ACG group). There was a significant reduction in IOP and medications in all three groups that lasted until the end of follow-up. Of note, there was a significant difference in the baseline IOPs between the groups, with the highest being in the solo group (25.37 ± 8.18, range 14–48 mmHg) and the lowest in the ACG group (16.26 ± 3.72, range 10–25 mmHg). The mean 24-month IOPs were 12.2 ± 2 (range 10–16 mmHg), 15 ± 6.7 (range 8–36 mmHg), and 14 ± 4.9 (range 9–36 mmHg) for ACG, solo OAG, and combined OAG, respectively. The greatest reduction in medication usage was in the combined group.

### 3.10. PostOperative Complications

Thirty eyes (23.8%) had an IOP spike, with a mean IOP of 37.2 ± 9.1 mmHg at presentation (range 23–68 mmHg). The mean duration of a spike was 6.3 ± 9.3 days. On average, the spike was diagnosed on postoperative day 8.8 ± 7.4 (range 1–72 days).

Hyphema was classified as microhyphema (red blood cells (RBCs) in the anterior chamber) or macrohyphema (blood or blood clot in the anterior chamber with or without RBCs). Eighty-one (63.3%) eyes were diagnosed with microhyphema, with a mean duration of 4.93 ± 6.7 days (1–41 days). Sixty eyes (46.9%) were diagnosed with macrohyphema, with a mean duration of 1.9 ± 3.7 days (1–27 days). Reduced visual acuity to the level of counting fingers or worse due to hyphema was documented in 15 eyes (11.7%), all of which improved after spontaneous resorption of the blood. Three patients (2.3%) had numerical hypotony, which was diagnosed on the first postoperative day in all three eyes and resolved spontaneously after 54, 14, and 10 days. Postoperative complications are shown in Table 5.

## 4. Discussion

The results of this retrospective study demonstrate good surgical success rates for GATT across a wide range of glaucomas in a diverse population in combination with lens extraction or as a standalone procedure. These results are in line with the current literature on GATT [23] which has been accumulating since the initial report by Grover et al. [1].

At 2 years, success was 72.5% for criterion A and 52% for criterion B.

IOP at 2 years was 15 mmHg, 14 mmHg, and 12.1 mmHg on 2, 0.9, and 1.4 medications for solo GATT, combined OAG, and ACG groups, respectively.

Twelve eyes (9.5%) needed re-operation for glaucoma by the end of follow-up.

Using criterion B, 55 eyes (43%) in our cohort were classified as failures, with 10% failing due to an IOP > 21 mmHg at the last follow-up. The higher failure rate under criterion B compared to criterion A can be explained by several factors. First, criterion B did not account for medication reduction. Second, most failures were attributed to a reduction of less than 20% from baseline IOP (53 eyes), which was primarily due to a lower baseline IOP—43 eyes in the cohort had a preoperative IOP of less than 20 mmHg.

Complete success, i.e., achieving specified IOP targets without medication, was not set as an outcome in this study. The reason for this stems from the difference between GATT and traditional filtration surgeries (especially trabeculectomy). Contrary to trabeculectomy, where drops are not routinely added in the first months after surgery and rather, digital massage, suture lysis, and/or bleb needling are used to modify flow, GATT surgeries often require adding medication in the early postoperative period to gain better IOP control, and these are then continued as needed [8].

Smith et al., over 24 months of follow-up, showed an average IOP decrease of 37.3% and 49.8% and an average glaucoma medication decrease of 1.43 and 2.0 in POAG and SOAG, respectively, in 198 patients [6]. Better efficacy of GATT in SOAG as compared to POAG was found in our study as well: a decrease of 27% in IOP and 2 in medications in POAG versus 35.4% in IOP and 2.2 in medications in SOAG.

Sharkawi et al. demonstrated good efficacy of GATT in ACG at 24 months with an IOP at final FU of 12.1 mmHg [7]. We found similar results with IOP reduction to 12.1 mmHg at 24 months. The number of medications was higher in our study (1.4 vs. 0.8) than in that by Sharkawi, possibly owing to differences in glaucoma severity and individually set target pressures. All cases of GATT in ACG were combined with cataract surgery. It is well known that lens removal alone is effective in IOP lowering in these cases [24,25] the addition of GATT to phacoemulsification in PACG is promising, with favorable outcomes in terms of IOP, glaucoma medications, and surgical success [26]. Aktas et al. retrospectively compared the outcomes of GATT between POAG and PXG patients. They reported a decrease of 8.8 mmHg (34.4%) and 12.8 (44.6%) in the POAG and PXG groups, respectively. Cumulative success at 1 year in POAG was 86.8% [23] similar to our POAG results after 1 year.

The decline in surgical success rates from year 1 to year 2 in our study is in line with current research. In the study by Wan et al. [27] the 1-year success rate for GATT combined with phacoemulsification was 86.21%, and for GATT alone, it was 83.48%. By 2 years, these rates declined to 65.0% and 55.5%, respectively. Similarly, the study by Pereira et al. reported a significant drop in success rates over time, with a 1-year relative success rate of 88.9% and a 2-year success rate of 43.9% [28].

Patients with controlled glaucoma who underwent cataract surgery combined with GATT were analyzed separately (Figure 3A). This group enjoyed IOP reduction, even though mean preoperative IOP was 15 mmHg. Importantly, medication was reduced by over 50%, suggesting a role for the addition of GATT to cataract surgery to decrease medication burden even in well-controlled patients undergoing cataract surgery. Medication reduction can have powerful benefits in terms of quality of life, compliance, diurnal IOP control, and disease progression.

Worse preoperative BCDVA and postoperative IOP spikes were found to be risk factors for failure to achieve criterion A in the multivariate analysis. Worse preoperative BCDVA may imply worse glaucoma severity at baseline.

Risk factors for failure requiring reoperation for glaucoma were analyzed separately. The reason for focusing on this subset of failures is that many of the failures in the study, though classified as failures by our stringent success criteria, were clinical successes. An example is a patient with ocular hypertension and a preoperative IOP of 32 on 3 medications who postoperatively had an IOP of 19 without medications. This patient is a failure by our criteria because the IOP is above 18, but he is a clinical success. By looking at patients who required re-operation we attempt to gain insight as to the characteristics of those patients in whom GATT may not have been the ideal surgical choice. High maximal IOP before surgery was a risk factor for failure, as was an IOP spike in the early postoperative period. It is unclear whether peak IOP is indicative of the exact anatomic location and/or extent of outflow obstruction. It has been shown that larger portions of Schlemm’s canal (SC) are collapsed in glaucomatous, as compared to normal eyes [29]. Also, changes in juxtacanalicular tissue, as well as increased stiffness of trabecular meshwork (TM) and SC cells have been documented in glaucomatous eyes [29,30,31].

It is possible that especially high IOPs may be an underestimated sign of the health of the above tissues and, perhaps even predictive of postoperative flow and IOP. This area requires further study.

Our data do not allow us to draw definitive conclusions about specific glaucoma subtypes in which GATT may be less effective. However, based on surgical principles and existing literature, GATT should be used with caution in cases with longstanding synechial angle closure, especially if these are related to intraocular inflammation, neovascular glaucoma, eyes with silicone oil, and eyes with abnormal angle anatomy.

The most common complications of surgery were hyphemas and IOP spikes. Sixty eyes (46.9%) were diagnosed with macrohyphema. Seuthe et al. reported occurrence of hyphema in the first 2 days in 22 cases (25%) [32]. Our rate was almost double, though this is likely due to different definitions. All hyphemas cleared spontaneously in both studies. Three eyes had numerical hypotony, which resolved spontaneously after 54, 14, and 10 days. Postoperative hypotony is uncommon after GATT but has been reported. The causes of hypotony are likely cyclodialysis clefts and ciliary shutdown [11,33]. IOP spikes were seen, on average, on day 8.8 ± 7.4 and lasted for a mean of 6.3 ± 9.3 days. The duration of the spikes reported in this study is an overestimation because of how it was measured. A spike was recorded as resolved on the first visit when the IOP normalized, though it was longer than the real duration of the spike in nearly all cases. In a previous study by our group, IOP spikes were diagnosed at a mean of 7.7 ± 6.5 days after surgery, and the mean duration of a spike was 4.9 ± 5.4 days [34]. Reoperation risk was strongly associated with IOP spikes, emphasizing the need for early spike management. A recent study analyzing the four-year surgical outcomes of GATT in OAG also reported IOP spikes as a risk factor for failure [35].

Patients were seen 4 times in the first 2 weeks after surgery and 5–6 times in the first month to catch spikes. IOP-lowering medications were routinely stopped after surgery for the study population, and spikes were treated aggressively with topical, oral, or intravenous medications, as needed. The authors have since changed their practice to continue at least some of the medications postoperatively in almost all patients and then taper them according to the individual clinical scenario.

This study has several important limitations. Its retrospective nature introduces inherent biases and potential confounders, such as glaucoma severity, which were not explicitly controlled for. This may affect the interpretation of the results. However, we employed multiple statistical methods, including multivariate logistic regression, Kaplan–Meier survival analysis, and Bonferroni correction for multiple comparisons, ensuring that statistical assumptions were met. Additionally, the study population was diverse in terms of demographics and ophthalmic conditions, and the extent of trabeculotomy was left to the discretion of the surgeon. While this variability represents a limitation, it also enhances the generalizability of our findings. Future prospective studies with larger sample sizes are needed to validate and further refine these results.

## 5. Conclusions

GATT is an effective surgical strategy for reducing IOP and medications in open- and closed-angle glaucoma, with and without concomitant cataract extraction. Risk factors for failure are worse baseline VA and postoperative IOP spike. Risk factors for re-operation are high maximal preoperative IOPs and postoperative IOP spikes. GATT should be considered early in the treatment paradigm of medically uncontrolled glaucoma, as well as in patients with controlled glaucoma undergoing cataract surgery. Further studies are needed to clarify which patients stand to benefit the most from this procedure.

## Figures and Tables

**Table 1:** Basic characteristics.

Parameter	Value	
Total Patients	126	
Demographics		
Eye, OD, *n*%	59 (46.8%)	
Age, years, Mean ± SD	68.9 ± 14.4	
Gender, Male, *n*%	73 (57.9%)	
Procedure		
Goniotomy, Angles, *n*%	180°	50 (39.7%)
	360°	38 (30.2%)
	200°	13 (10.3%)
	270°	18 (14.3%)
	90°	7 (5.6%)
Hemi-GATT, *n*%	Inferior	41 (65.1%)
	Superior	22 (34.9%)
Procedure Type, *n*%	Phaco + GATT	87 (69.1%)
	Solo GATT	28 (22.2%)
	Phaco + GSL + GATT	5 (4.0%)
	GATT + IOL fixation	6 (4.8%)
Preoperative data		
Glaucoma Type, *n*%	POAG	31 (24.6%)
	SOAG	77 (61.1%)
	PACG	18 (14.3%)
Glaucoma Severity, *n*%	Mild	36 (28.6%)
	Moderate	27 (21.4%)
	Advanced	49 (38.9%)
	OHTN	14 (11.1%)
Vertical C/D ratio, Mean ± SD	0.70 ± 0.23	
Visual Field, MD (dB), Mean ± SD	−9.17 ± 6.68	
LogMAR (VA), Mean ± SD	0.48 ± 0.50	
IOP (last before surgery), mmHg, Mean ± SD	20.65 ± 7.28	
Number of medications, Mean ± SD	3.47 ± 1.54	
Oral CAI Usage, *n*%	38 (30.2%)	
Post-op Data		
Maximal IOP, mmHg, Mean ± SD	33.3 ± 10.5	
PO Duration, days, Mean ± SD	583 ± 266	
Last IOP, mmHg, Mean ± SD	14.21 ± 6.41	
Number of medications, last visit, Mean ± SD	1.40 ± 1.52	
Oral CAI usage at last visit, *n*%	8 (6.4%)	
Last PO logMAR VA, Mean ± SD	0.31 ± 0.52	

Abbreviations: OD (oculus dexter, right eye), SD (standard deviation), GATT (goniotomy-assisted transluminal trabeculotomy), Phaco + GATT (phacoemulsification + goniotomy-assisted transluminal trabeculotomy), SOAG (secondary open-angle glaucoma), POAG (primary open-angle glaucoma), PACG (primary angle-closure glaucoma), OHTN (ocular hypertension), C/D (cup-to-disc ratio), MD (mean deviation, visual field), LogMAR (logarithm of the minimum angle of resolution, VA (visual acuity), IOP (intraocular pressure), CAI (carbonic anhydrase inhibitor), PO (postoperative), IOL (intraocular lens), POD (post-operative day).

**Table 2:** GATT failures and reoperations.

Failures Criterion A and Reoperations	
Total number, *n* (%)	44 (34.0%)
IOP > 18, *n* (%)	22 (17.4%)
Higher IOP than baseline, *n* (%)	26 (20.6%)
More medications than baseline, *n* (%)	6 (4.7%)
Reoperations	
Total number, *n* (%)	12 (9.5%)
AGV, *n* (%)	8 (6.3%)
Trabeculectomy, *n* (%)	4 (3.1%)

Abbreviations: IOP—intraocular pressure, AGV—Ahmed glaucoma valve.

**Table 3:** GATT outcomes by glaucoma type.

Glaucoma Type	Total Eyes (*n*)	Failures, *n* (%)		Success, *n* (%)		Mean Pre-op IOP (mmHg)	95% CI	Mean PO IOP (mmHg)	95% CI	ΔIOP (mmHg)	% IOP Reduction	Mean Pre-op Meds	95% CI	Mean PO Meds	95% CI	ΔMeds	% Med Reduction
**POAG**	31	14	45.2%	17	54.8%	19.35	16.83–21.88	15.65	13.88–17.41	3.70	19.1%	3.32	2.81–3.84	1.48	0.84–2.13	1.84	55.4%
**PXG**	36	10	27.8%	26	72.2%	22.56	20.04–25.08	13.89	12.19–15.58	8.67	38.4%	3.94	3.48–4.41	1.37	0.88–1.86	2.57	65.2%
**PACG**	18	9	50.0%	9	50.0%	16.50	14.78–18.22	15.59	11.49–19.68	0.91	5.5%	3.17	2.61–3.72	1.61	0.77–2.45	1.56	49.1%
**CMG**	15	5	26.7%	10	73.3%	21.40	17.77–25.03	14.57	10.90–18.24	6.83	31.9%	2.93	2.21–3.66	1.79	1.33–2.40	1.15	39.1%
**OHTN**	12	3	25.0%	9	75.0%	22.67	17.37–27.96	12.55	10.65–14.44	10.12	44.6%	3.17	2.04–4.29	0.00		3.17	100.0%

**Table 4:** Efficacy data for study population, separated by study group.

Time	*n*	Mean Pre-Op IOP (mmHg)	95% CI	Mean PO IOP (mmHg)	95% CI	ΔIOP (mmHg)	% IOP Reduction	Mean Pre-Op Meds	95% CI	Mean PO Meds	95% CI	ΔMeds	% Med Reduction
OAG combined phaco+GATT													
1 D	70	19.70	(18.1–21.2)	15.53	(13.8–17.2)	4.17	21.2%	3.03	(2.75–3.31)	0.31	(0.14–0.48)	2.71	89.6%
1 W	69	19.43	(17.8–21)	17.01	(15.5–18.5)	2.42	12.4%	3.01	(2.7–3.2)	0.50	(0.3–0.7)	2.51	83.4%
3 W	62	19.84	(18.1–21.5)	15.68	(14.2–17)	4.16	21.0%	3.06	(2.7–3.3)	0.94	(0.6–1.2)	2.13	69.4%
6 W	57	20.02	(18.2–21.8)	13.42	(12.4–14.3)	6.60	32.9%	3.00	(2.6–3.3)	0.93	(0.6–1.2)	2.07	69.0%
3 M	59	20.00	(18.3–21.7)	13.90	(12.8–14.9)	6.10	30.5%	3.30	(2.9–3.7)	0.88	(0.58–1.17)	2.42	73.3%
6 M	53	19.72	(18–21.3)	13.42	(12–14.7)	6.30	31.9%	3.09	(2.8–3.4)	0.89	(0.6–1.2)	2.21	71.3%
9 M	51	19.33	(17.5–21.2)	12.55	(11.8–13.2)	6.78	35.1%	3.10	(2.7–3.4)	0.69	(0.4–0.9)	2.41	77.8%
12 M	54	19.30	(17.5–21.1)	13.50	(12.2–14.8)	5.80	30.1%	3.40	(2.9–3.8)	1.00	(0.7–1.4)	2.40	70.6%
24 M	52	19.7	(17.9–21.6)	14.00	(12.6–15.4)	5.7	28.9%	3.25	(2.8–3.6)	0.9	(0.5–1.3)	2.35	72.3%
OAG solo GATT													
1 D	29	25.38	(22.3–28.3)	13.00	(10.8–15.1)	12.38	48.8%	3.93	(3.6–4.2)	0.66	(0.2–1.1)	3.28	83.3%
1 W	28	25.61	(22.5–28.6)	18.57	(15.5–21.5)	7.04	27.5%	3.96	(3.6–4.3)	1.00	(0.4–1.6)	2.96	74.7%
3 W	28	25.61	(22.5–28.6)	18.54	(14.1–22.9)	7.07	27.6%	3.96	(3.6–4.3)	1.54	(0.9–2.1)	2.43	61.3%
6 W	23	25.61	(22–29.2)	14.04	(10.8–17.2)	11.57	45.2%	4.04	(3.7–4.4)	1.87	(1.2–2.5)	2.17	53.8%
3 M	28	25.21	(22.1–28.3)	12.79	(11.2–14.3)	12.43	49.3%	3.93	(3.5–4.2)	1.64	(1–2.2)	2.29	58.2%
6 M	26	25.08	(21.8–28.2)	13.65	(11.8–15.4)	11.42	45.5%	3.96	(3.6–4.3)	1.62	(1–2.2)	2.35	59.2%
9 M	21	26.52	(22.7–30.30)	13.76	(11.6–16)	12.76	48.1%	3.81	(3.4–4.2)	1.95	(1–2.8)	1.86	48.7%
12 M	23	26.50	(22.3–30.6)	14.00	(11.6–16.4)	12.50	47.1%	4.30	(3.8–4.8)	1.60	(0.9–2.1)	2.70	62.8%
24 M	21	26.80	(21.8–31.8)	15.00	(11.5–18.6)	11.80	44.0%	4.40	(3.8–5)	2.20	(1.3–3)	2.20	50.0%
ACG combined phaco+GATT													
1 D	19	16.26	(14.6–17.9)	16.63	(13.8–19.4)	−0.37	−2.2%	3.11	(2.6–3.5)	0.68	(0.2–1.1)	2.42	78.0%
1 W	19	16.26	(14.6–17.9)	21.11	(15.2–27)	−4.84	−29.7%	3.11	(2.6–3.5)	0.79	(0.4–1.1)	2.32	74.5%
3 W	17	16.41	(14.8–18.2)	14.35	(12.2–16.5)	2.06	12.5%	3.00	(2.5–3.5)	1.24	(0.5–2)	1.76	58.8%
6 W	16	16.69	(14.8–18.5)	13.13	(11.9–14.3)	3.56	21.3%	3.06	(2.5–3.5)	0.94	(0.4–1.5)	2.13	69.4%
3 M	16	16.81	(15–18.5)	10.94	(9.8–12)	5.88	34.9%	3.19	(2.7–3.7)	1.06	(0.5–1.6)	2.13	66.7%
6 M	12	16.85	(14.7–18.9)	14.08	(11.4–16.7)	2.76	16.4%	3.23	(2.7–3.7)	1.08	(0.4–1.7)	2.15	66.5%
9 M	12	15.50	(13–17.9)	12.17	(10.9–13.4)	3.33	21.5%	3.00	(2.4–3.5)	0.92	(0.3–1.5)	2.08	69.4%
12 M	15	16.30	(14.3–18.3)	15.60	(12.5–18.7)	0.70	4.3%	3.20	(2.6–3.8)	1.40	(0.8–2.2)	1.80	56.2%
24 M	12	16.00	(14.2–17.7)	12.10	(10.9–13.4)	3.90	24.4%	3.50	(3–4.1)	1.40	(0.5–2.2)	2.10	60.0%

Abbreviations: OAG—open-angle glaucoma; ACG—angle-closure glaucoma; D—day; W—week; M—month; IOP—intraocular pressure; CI—confidence interval; meds—medications; pre-op—preoperative; PO—postoperative.

**Table 5:** Postoperative complications.

Complications		
IOP Spike	Total, *n* (%)	30 (23.8)
	POD of spike, days, Mean ± SD	8.8 ± 7.4
	Maximal IOP on spike, mmHg, Mean ± SD	37.2 ± 9.1
Numerical Hypotony, *n* (%)		3 (2.3%)
Microhyphema	Total, *n* (%)	81 (63.3%)
	Last day seen, days, Mean ± SD	4.9 ± 6.7
Macrohyphema	Total, *n* (%)	60 (46.9%)
	Last day seen, days, Mean ± SD	1.9 ± 3.7
VA, CF or worse due to hyphema, *n* (%)		15 (11.7%)

Abbreviations: IOP—intraocular pressure; VA—visual acuity; CF—counting fingers; SD—standard deviation.

## Data Availability

The data presented in this study are available on request from the corresponding author. the data are not publicly available due to patient confidentiality and institutional restrictions.

## Supplementary Materials

The following supporting information can be downloaded at: https://www.mdpi.com/article/10.3390/diagnostics15101226/s1.
