# Supplementary material for: Surgical Outcomes of Gonioscopy-Assisted Transluminal Trabeculotomy (GATT) in Primary and Secondary Open- and Closed-Angle Glaucoma
Source: Diagnostics (Basel). 2025 May 13;15(10):1226. doi: 10.3390/diagnostics15101226 (PMC12109802; doi:10.3390/diagnostics15101226)
Supplement: Supplementary file 1 [file diagnostics-15-01226-s001.zip › diagnostics-3481869-supplementary.pdf]

Supplementary Tables

Table S1. Risk factors for requiring additional glaucoma surgery.

Abbreviations: N-number; IOP- intraocular pressure; PO- post operative; POD1-post operative day 1; pre-op- pre operativ

Parameter		No surgery	Another surgery	*p*
**n**		114 (90%)	12 (10%)	
**Pre op Demographics**				
**Eye, OD , n(%)**		54 (47%)	5 (42%)	0.7
**Age, (mean±std)**		69.4±14.6	65.5±11.5	0.2
**Gender, male, n(%)**		67 (59%)	6 (50%)	0.6
**Pre-op loaMAR VA (mean±std)**		0.5±0.5	0.5±0.3	0.8
**Pre-op IOP last before surgery (mean±std)**		20.2±7.3	24.1±6.5	0.08
**Mean number of medications last IOP (mean±std)**		3.4±1.5	4.4±1.2	**0.01**
**Pre op oral CAI , n(%)**		31 (27%)	7 (58%)	**0.04**
**Maximal IOP, (mean±std)**		32.2±9.6	42.2±14	**0.003**
**Glaucoma type, n(%)**	POAG 1	27 (24%)	4 (33%)	0.5
	PXG 2	34 (30%)	2 (17%)	
	PDS 3	1 (1%)	0 (0%)	
	PACG 4	16 (14%)	2 (17%)	
	CMG 5	13 (11%)	2 (17%)	
	Uveitic 6	4 (4%)	1 (8%)	
	NTG 8	1 (1%)	0 (0%)	
	JOAG 10	1 (1%)	1 (8%)	
	OHTN 11	12 (11%)	0 (0%)	
	other 13	5 (4%)	0 (0%)	
**Glaucoma type, n(%)**	POAG	40 (35%)	5 (42%)	0.8

	SOAG	58 (51%)	5 (42%)	
	PACG	16 (14%)	2 (17%)	
**Glaucoma severity , n(%)**	mild	30 (26%)	2 (17%)	0.5
	moderate	23 (20%)	3 (25%)	
	advanced	42 (37%)	7 (58%)	
	OHTN	15 (13%)	0 (0%)	
**Procedure related parameters**				
**Procedure, n(%)**	Phaco+GATT	80 (70%)	6 (50%)	0.3
	solo GATT	23 (20%)	5 (42%)	
	phaco+GSL+GATT	4 (4%)	1 (8%)	
	GATT+IOL fixation	7 (6%)	0 (0%)	
**Trabeculotomy extent, n(%)**	180	41 (36%)	9 (75%)	**0.04**
	360	38 (33%)	0 (0%)	
	200	13 (11%)	0 (0%)	
	270	16 (14%)	2 (17%)	
	90	6 (5%)	1 (8%)	
**PO data**				
**PO last visit IOP (mean±std)**		13.3±5.4	22±9.7	**0**
**Mean number of medications last visit, (mean±std)**		1.1±1.3	3.7±1.6	**0**
**CAI PO last visit, n(%)**		1 (1%)	7 (58%)	**0**
**Last PO logMAR VA, (mean±std)**		0.27±0.4	0.7±1	**0.007**
**IOP POD1**		15.7±15.3	16.7±5.3	0.8
**IOP spikes, n(%)**		23 (20%)	7 (58%)	**0.008**
**Macro-hyphema, n(%)**		58 (51%)	4 (33%)	0.2
**Qualified suceess, n(%)**		82 (72%)	0 (0%)	**0**

Abbreviations: OD – oculus dexter (right eye); VA – visual acuity; logMAR – logarithm of the minimum angle of resolution; IOP – intraocular pressure; CAI – carbonic anhydrase inhibitor; POAG – primary open-angle glaucoma; PXG – pseudoexfoliation glaucoma; PDS – pigment dispersion syndrome; PACG – primary angle-closure glaucoma; CMG – combined mechanism glaucoma; NTG – normal-tension glaucoma; JOAG – juvenile open-angle glaucoma; OHTN – ocular hypertension; SOAG – secondary open-angle glaucoma; Phaco – phacoemulsification; GATT – gonioscopy-assisted transluminal trabeculotomy; GSL – goniosynechialysis; IOL – intraocular lens; POD1 – postoperative day 1; pre op – preoperative; PO – postoperative.

Table S2. Risk factors for surgical failure.

Parameter		Failure	Success	*p*
**n**		44 (35%)	82 (65%)	
**Pre op Demographics**				
**Eye, OD , n(%)**		19 (43%)	40 (49%)	0.6
**Age, (mean±std)**		67.8±13.3	69.5±15	0.5
**Gender, male, n(%)**		22 (50%)	51 (62%)	0.2
**Pre op loaMAR VA (mean±std)**		0.6±0.6	0.42±0.41	**0.05**
**Pre op IOP last before surgery (mean±std)**		20.2±8.5	20.8±6.5	0.6
**Mean number of medications last IOP (mean±std)**		3.5±1.6	3.5±1.5	0.9
**Pre op oral CAI , n(%)**		16 (36%)	22 (27%)	0.2
**Maximal IOP, (mean±std)**		34.5±12.7	32.7±9.2	0.4
**Glaucoma type, n(%)**	POAG 1	14 (32%)	17 (21%)	0.6
	PXG 2	10 (23%)	26 (32%)	
	PDS 3	0 (0%)	1 (1%)	
	PACG 4	9 (20%)	9 (11%)	
	CMG 5	5 (11%)	10 (12%)	
	Uveitic 6	1 (2%)	4 (5%)	
	NTG 8	0 (0%)	1 (1%)	
	JOAG 10	1 (2%)	1 (1%)	
	OHTN 11	3 (7%)	9 (11%)	
	other 13	1 (2%)	4 (5%)	0.1
	POAG	18 (41%)	27 (33%)	
	SOAG	17 (39%)	46 (56%)	
	PACG	9 (20%)	9 (11%)	
**Glaucoma severity , n(%)**	mild	11 (25%)	21 (26%)	0.3
	moderate	11 (25%)	15 (18%)	

	advanced	20 (45%)	29 (35%)	
	OHTN	2 (5%)	13 (16%)	
**Procedure related parameters**				
**Procedure, n(%)**	Phaco+GATT	30 (68%)	56 (68%)	0.9
	solo GATT	10 (23%)	18 (22%)	
	phaco+GSL+GATT	2 (5%)	3 (4%)	
	GATT+IOL fixation	2 (5%)	5 (6%)	
**Trabeculotomy extent, n(%)**	180	21 (48%)	29 (35%)	0.5
	360	9 (20%)	29 (35%)	
	200	4 (9%)	9 (11%)	
	270	7 (16%)	11 (13%)	
	90	3 (7%)	4 (5%)	
**PO data**				
**PO last visit IOP (mean±std)**		19.3±7.2	11.4±3.7	**0**
**Mean number of medications last visit, (mean±std)**		2.2±1.7	0.9±1.1	**0**
**CAI PO last visit, n(%)**		8 (18%)	0 (0%)	**0**
**Last PO logMAR VA, (mean±std)**		0.4±0.7	0.3±0.4	0.3
**IOP POD1**		15.3±5.7	16±17.8	0.8
**IOP spikes, n(%)**		15 (34%)	15 (18%)	**0.05**
**Macro-hyphema, n(%)**		21 (48%)	41 (50%)	0.8
**Rescue surgery, n(%)**		12 (27%)	0 (0%)	**0**

Abbreviations: OD – oculus dexter (right eye); VA – visual acuity; logMAR – logarithm of the minimum angle of resolution; IOP – intraocular pressure; CAI – carbonic anhydrase inhibitor; POAG – primary open-angle glaucoma; PXG – pseudoexfoliation glaucoma; PDS – pigment dispersion syndrome; PACG – primary angle-closure glaucoma; CMG – combined mechanism glaucoma; NTG – normal-tension glaucoma; JOAG – juvenile open-angle glaucoma; OHTN – ocular hypertension; SOAG – secondary open-angle glaucoma; Phaco – phacoemulsification; GATT – gonioscopy-assisted transluminal trabeculotomy; GSL – goniosynechialysis; IOL – intraocular lens; POD1 – postoperative day 1; pre op – preoperative; PO – postoperative.
